# Association Between Sleep Quality and Duration During Pregnancy and Risk of Infant Being Small for Gestational Age: Prospective Birth Cohort Study

**DOI:** 10.3390/healthcare12232400

**Published:** 2024-11-29

**Authors:** Huimin Zhu, Xinchen Liu, Min Wei, Rui Gao, Xuemei Liu, Xiuxiu Li, Xuhua Liu, Weiqing Chen

**Affiliations:** 1 Department of Epidemiology, School of Public Health, Sun Yat-sen University, Guangzhou 510080, China; zhuhm9@mail2.sysu.edu.cn (H.Z.); liuxch57@mail2.sysu.edu.cn (X.L.); 2 Department of Science and Education, Shenzhen Birth Cohort Study Center, Nanshan Maternity and Child Healthcare, Shenzhen 518067, China; minwei@szbcs.org (M.W.); ruigao@szbcs.org (R.G.); liuxm@szbcs.org (X.L.); lixx@szbcs.org (X.L.); liuxh@szbcs.org (X.L.); 3 Shenzhen Cadre and Talent Health Institute (Shenzhen Talent Institute), Shenzhen 518071, China

**Keywords:** pregnancy, sleep, small for gestational age, critical window, birth cohort study

## Abstract

Background: Maternal sleep disturbance is a risk factor for adverse outcomes like preterm birth. However, the association of maternal sleep quality and duration with the risk of the infant being small for gestational age (SGA) remains inconclusive, and the specific critical window of vulnerability has yet to be clearly identified. Therefore, this study aims to investigate the effect of maternal sleep quality and duration on the risk of having an SGA infant and to identify the critical window for this association. Methods: One thousand six hundred and seventy-seven participants from the Shenzhen Birth Cohort Study were included. Maternal sleep duration and quality during pregnancy were assessed using the Pittsburgh Sleep Quality Index (PSQI) in early (<19 weeks), mid- (24–28 weeks), and late (32–38 weeks) pregnancy. Multivariate logistic regression analyses were used to examine the association of an SGA infant with sleep duration and quality, along with their specific effects across the different pregnancy stages. Results: The pregnant women with short sleep duration (≤7 h/day) in the early stage of pregnancy appeared to have a higher risk of having an SGA infant (aOR = 1.93, 95% CI = 1.32~2.79). Additionally, poor sleep quality combined with short sleep duration was associated with an even higher risk of having an SGA infant (aOR = 2.08, 95% CI = 1.32~3.23). However, this association was observed only during early pregnancy. Conclusions: The women with short sleep duration were associated with SGA risk, and the early stage of pregnancy might be a particularly sensitive period for this relationship. Addressing maternal sleep problems during pregnancy as part of antenatal care is crucial for reducing the likelihood of having an SGA infant and improving the overall birth outcomes.

## 1. Introduction

Small for gestational age (SGA), which is defined as an infant having an estimated fetal weight (EFW) or birth weight (BW) below the 10th percentile for their gestational age, is commonly used to suspect fetal growth restriction [1]. In 2020, 17.4% of live infants (23.4 million) were born SGA, with 40.9% in southern Asia. In low- and middle-income countries, over 20% of neonatal deaths were attributable to SGA [2]. Additionally, SGA neonates are not only at a higher risk of neonatal mortality and morbidity but are also associated with poor neurodevelopment and long-term metabolic syndrome later in life [3,4,5]. Accordingly, SGA remains a significant global public health concern.

Sleep disturbances characterized by abnormal sleep quantity and quality, as well as specific disorders, such as sleep-disordered breathing (SDB) and insomnia, are common complaints among pregnant women due to the physiological and psychological changes that occur during pregnancy [6,7,8]. The emerging evidence suggests that sleep disorders during pregnancy are significant risk factors for adverse maternal and infant birth outcomes, including preeclampsia, gestational diabetes mellitus, and preterm birth (PTB) [9,10]. It has also been indicated that SDB or obstructive sleep apnea (OSA) during pregnancy is associated with SGA [7,11]. However, research on the relationship of maternal sleep quality and duration during pregnancy with SGA is limited, and the findings are controversial and inconclusive. A prior cross-sectional study indicated that sleep quality could influence neonates’ birth weight [12]. In a prospective cohort study, Abeysena et al. found that maternal sleep deprivation in late pregnancy is a risk factor for severe SGA (less than the fifth percentile) [13]. However, another study by Micheli et al. showed no association between sleep deprivation and restricted fetal growth [14].

Recently, a growing body of research has focused on identifying the critical window for prenatal exposure to adverse health outcomes. For instance, prenatal exposure to particulate matter < 2.5 μm has been associated with adverse birth outcomes, with varying sensitive windows [15]. Zhang et al. reported that the third trimester is a critical period for pregnancy-related anxiety, which influences children’s emotional and behavioral development [16]. However, only limited studies have examined the critical window for maternal sleep disorders and SGA. In this prospective cohort study, we aim to investigate the effect of maternal sleep quality and duration at different stages of pregnancy on the risk of having an SGA infant and explore whether the different stages of pregnancy may represent critical windows for this association.

## 2. Materials and Methods

### 2.1. Study Population

The Shenzhen Birth Cohort Study (SZBCS, NCT03830879) is population-based and prospective and was conducted in Shenzhen Nanshan Maternity and Child Healthcare Hospital. This study was designed to explore the influence of environmental and genetic factors on fetal growth, birth outcomes, and childhood development. The participants were recruited before 19 weeks of gestation and received antenatal and child healthcare at this hospital. During pregnancy, the participants attended three follow-up visits (<19 weeks of pregnancy at recruitment, 24–28 weeks of pregnancy, and 32–38 weeks of pregnancy), during which they completed a questionnaire that requested socio-demographic information, their medical history, lifestyle data (including those related to physical activity, sleep quality and quantity, and food preferences and habits), and environmental details. Meanwhile, clinical examinations were conducted, and simultaneously biological samples were collected. After delivery, the birth outcomes were recorded, and the childhood follow-up was initialed, including growth scans, biological sample collection, and questionnaires about child nurturing. Informed consent was obtained from all the participants. The procedures and methods were approved by the ethics committees at Nanshan Maternity and Child Healthcare Hospital of Shenzhen (NSFYEC-KY-2020031) and Sun Yat-sen University (2018–054).

This study was derived from the SZBCS data. Initially, 3811 participants were enrolled in the SZBCS from January 2018 to December 2022, and 2837 of them met the inclusion criteria by completing questionnaires during early, mid-, and late pregnancy. After excluding 303 participants who delivered large-for-gestational-age (LGA) neonates, 45 participants with multiple gestations, 9 participants with fetal malformations, 18 participants with fetal cessation of development, 93 participants who experienced an abortion, 642 participants who did not provide baseline or birth information, and 50 participants with PTB, 1677 participants were finally included in this study.

The sample size was calculated using a method for cohort studies that accounts for the overall prevalence of SGA in China and populations with maternal short sleep duration during pregnancy [17], assuming a power level of 90% and a 2-sided α of 0.05. A sample size of 296 participants in each group was calculated. Further, assuming a loss of follow-up of 20% led to a minimum sample size of 356 participants for each group.

### 2.2. Assessment of Sleep Quality and Duration During Pregnancy

The sleep characteristics were assessed at each antenatal care visit (<19 weeks, 24–28 weeks, 32–38 weeks of pregnancy) using the Pittsburgh Sleep Quality Index (PSQI), which has demonstrated adequate validity and reliability [18,19], reflecting sleep quality over the previous month. The PSQI includes 18 items divided into 7 sleep-related subscales: subjective quality, latency, duration, habitual sleep efficiency, sleep disturbance, use of sleeping medications, and daytime dysfunction. Each subscale was scored from 0 to 3, and the PSQI global score ranged from 0 to 21. A higher score reflects poorer sleep quality, and a global PSQI score > 5 is the cut-off point indicating poor sleep quality. Sleep duration was assessed using the following PSQI question, ‘How long did you actually fall asleep per night over the past month?’, and then the participants were categorized into short [≤7 h] and normal sleep duration (>7 h) groups in this study.

### 2.3. Birth Outcomes

The birth outcomes included the neonates’ gestational age at the time of delivery (week), gender, and BW. The neonates with a BW < 10th percentile or >90th percentile according to a population-based gender-specific reference were classified as being SGA and LGA, respectively. The newborns with BW > 10th percentile and <90th percentile were classified as being appropriate for gestational age (AGA) in this study [20].

### 2.4. Covariates

Information on the demographic characteristics, health status, and lifestyle factors of the pregnant women was collected by trained staff through a self-report questionnaire during the first visit. The maternal demographic characteristics included age, education status, marital status (married or unmarried/widowed), income level (<5000, 5000–10,000, 10,000–15,000, or >15,000 in CNY), and employment status (employed or unemployed). Health status included their pre-pregnancy body mass index (BMI), conception method (natural or use of assisted reproductive technology), parity (primiparous or multiparous), and medical history (including gestational hypertension and gestational diabetes mellitus). Pre-pregnancy BMI (calculated as weight in kilograms divided by height in meters squared) was categorized according to the Chinese standard into underweight (BMI < 18.5 kg/m^2^), normal weight (BMI 18.5–23.9 kg/m^2^), and overweight/obesity (BMI ≥ 24 kg/m^2^) [21]. The lifestyle factors included whether they smoked or consumed alcohol before pregnancy (yes or no).

The potential confounders were identified using a directed acyclic graph (DAG) and change-in-estimate (CIE) analysis [22,23]. Briefly, we created a DAG (Figure S1) to determine the minimal sufficient adjustment set of variables, which were subsequently included in the multivariate logistic regression model. The covariates were retained as potential confounders if they changed the estimated effect of maternal sleep disturbance on SGA by more than 10%. According to these strategies, maternal age, pre-pregnancy BMI, and parity were identified as potential confounders in final analysis.

### 2.5. Statistical Analysis

Based on the distribution of the data, which was either normal or skewed, means (standard deviation, SD) or medians (quartiles) were used to describe the continuous variables, while absolute frequencies and proportions were used to describe the categorical and ordinal variables. The continuous variables were compared using Student’s *t*-test or Kruskal–Wallis test depending on their distribution, while the categorical and ordinal variables were compared with a chi-squared test.

Univariate and multivariate logistic regression models were used to estimate the association between poor sleep quality and short sleep duration during pregnancy and the risk of having an SGA infant, as well as their combined effects at each stage. Additionally, considering one of the PSQI components was subjective sleep quality, we also estimated the contribution of the individual component on the risk of having an SGA infant. In the multivariate logistic regression model, the odds ratio was adjusted for potential confounders, including maternal age, pre-pregnancy BMI, and parity. Cross-over analysis [24] was also used to estimate the stage-specific effects of sleep quality and duration on SGA risk using good sleep quality or normal sleep duration throughout pregnancy as the reference group. Prior to cross-over analysis, two-way repeated measures analysis of variance was conducted to determine whether the impact of sleep disturbance was independent at each stage, and the results showed that the interaction term was not statistically significant. Given the reported sex-specific effects of sleep disorders [25], main analysis was also repeated by sex.

*p* value < 0.05 (two-sided) was considered to be statistically significant. All analyses were performed using R, version 4.2.2.

## 3. Results

### 3.1. Baseline Characteristics

A total of 1677 pregnant women were included in this study, with a median age of 30.5 (4.1) years. Among the participants, 86.5% had a college or university education or higher, and 88.0% were employed. The prevalence of SGA was 8.5%. The pregnant women who delivered SGA neonates had a higher proportion of being underweight and primiparous compared to that of those who delivered AGA neonates (Table 1).

### 3.2. Effect of Maternal Sleep Quality and Duration in Different Stage of Pregnancy on SGA

Compared with the mothers who had good sleep quality, those who had poor sleep quality in early, mid-, and late pregnancy had elevated risks of SGA, with adjusted ORs of 1.29 (95% CI = 0.91~1.83), 1.06 (95% CI = 0.75~1.51), and 1.14 (95% CI = 0.80~1.61), respectively, though these associations were not statistically significance (Table 2). Additional analysis (Figure S2) on the PSQI component also showed no significant association between subjective sleep quality and SGA risk (aOR = 1.06, 95% CI = 0.81~1.37).

Compared to the pregnant women with normal sleep duration, those with short sleep duration in early pregnancy had a significantly higher risk of having an SGA infant (aOR = 1.93, 95% CI = 1.32~2.79). Furthermore, in early pregnancy, the risk for those with sleep duration allocated into second, third, and fourth quartiles was lower when compared to the risk for those allocated into first quantile (*p* trend = 0.009) (Table S2). For those with short sleep duration in mid- (aOR = 0.86, 95% CI = 0.59~1.28) and late pregnancy (aOR = 0.92, 95% CI = 0.64~1.33), the risks of SGA were not increased.

Further stratification analysis showed that only the pregnant women with both poor sleep quality and short sleep duration in early pregnancy had a significantly higher risk of having an SGA infant (aOR = 2.08, 95% CI = 1.32~3.23) (Table 3).

### 3.3. Effect of Different Combinations of Maternal Short Sleep Duration in Three Stages of Pregnancy on SGA

Table 4 illustrates the risk of having an SGA infant for women with short sleep duration in the early, mid-, and late stages of pregnancy, presented in seven categories. Though there is no statistical significance, except for the mothers with short sleep duration during the entire pregnancy, those with a pattern that involved short sleep duration in early pregnancy all exhibited higher risks of SGA, with adjusted ORs of 1.38 (95% CI = 0.62~2.78), 2.08 (95% CI = 0.95~4.17), 1.92 (95% CI = 0.91~3.72), and 1.82 (95% CI = 1.02~3.13) compared with those of the mothers who had normal sleep duration throughout pregnancy.

### 3.4. Sex-Specific Effect of Maternal Short Sleep Duration in Three Stages of Pregnancy on SGA

As shown in Table 5, in early pregnancy, the effect of maternal sleep quality and duration on SGA infant boys was significantly greater, with adjusted ORs of 1.79 (95% CI = 1.10~2.96) and 2.73 (95% CI = 1.63~4.56), respectively. In contrast, this effect was not significant among girls, with an adjusted OR of 0.94 (95% CI = 0.55~1.56) for maternal sleep quality and 1.33 (95% CI = 0.73~2.33) for duration.

## 4. Discussion

In this prospective birth cohort study, we found that the pregnant women with short sleep duration in the early stage of pregnancy had a significantly higher risk of having an SGA infant. Further stratification analysis indicated that those with a combination of poor quality and short sleep duration in the early stage of pregnancy had an even higher risk of having an SGA infant. Cross-over analysis also indicated that pregnant women with the pattern involving short sleep duration in the early stage of pregnancy all exhibited an elevated risk of having an SGA infant, though this was not statistically significant. Subgroup analysis showed that the effects of sleep quality and duration in early pregnancy were significant among boys but not among girls.

### 4.1. Associations of Maternal Sleep Duration and Quality During Pregnancy with the Risk of Having an SGA Infant

In this study, we found that the pregnant women with short sleep duration in early pregnancy had a higher risk of having an SGA infant (aOR = 1.93, 95%CI = 1.32~2.79). However, no significant effect was observed for maternal sleep duration during the mid- and late stages of pregnancy or for maternal sleep quality (including subjective sleep quality) at any stage of pregnancy on the risk of having an SGA infant. We also found that the pregnant women with both short sleep duration and poor sleep quality in the early stage of pregnancy had an even much higher risk of having an SGA infant (aOR = 2.08, 95% CI = 1.32~3.23). In recent decades, there have been several studies on associations between maternal sleep duration and quality in pregnancy and fetal growth, but the results are inconsistent. For instance, a cohort study of 690 pregnant women in Sri Lanka reported that sleeping for 8 h or less during the second and/or third trimesters of pregnancy was a risk factor for having an SGA infant below the fifth percentile [13]. A prospective cohort study in Chengdu, China, found that pregnant women with short sleep duration (average nightly sleep ≤ 7 h) during the third trimester of pregnancy were more likely to deliver SGA infants (OR = 2.67, 95% = 1.18~6.54) [26]. Qian Yang et al.’s study indicated that the risk of low birth weight was higher among European pregnant women who reported sleeping ≤ 5 h/d or ≥10 h/d compared with the risk for those with a sleep duration of 8–9 h/d [27]. In contrast, a prospective cohort study in Wuhan, China, found that, compared to those who had sleep duration from 8 to <9 h/per day, sleeping for <7 h/day resulted in a decrease of 42.70 g in the infants’ birth weight, and the risks of LBW and SGA increased by 83% and 56%, respectively, though these association were not statistically significant [28]. Additionally, another cohort study in Japan did not find a significant association between maternal sleep quality or duration during pregnancy and the risk of having an SGA infant at birth [29]. In addition to sleep duration, pregnant women with low-quality sleep during the third trimester of pregnancy had a higher risk of delivering SGA infants (OR = 2.67, 95% = 1.18~6.54) compared with the risk for those with good quality. Overall, the inconsistent findings across the studies mentioned above may be attributed to the differences in the trimester examined and geographical location, sociocultural backgrounds, data collection methods, and the control of potential confounding variables.

According to the critical period model theory of life course epidemiology, a critical window refers to a limited time period during which exposure could have adverse or protective effects on development and subsequent disease or disorder with no excess disease risk associated with exposure outside this time period [30,31]. Indeed, the emerging evidence has demonstrated that pregnancy is a critical window for prenatal environmental exposure, which can lead to adverse health outcomes at birth or later in life [32,33,34,35]. However, research exploring the critical window for the effect of sleep disturbance on birth outcomes is limited. A case–control study containing 479 women indicated that less sleep in the first six months of pregnancy was significantly associated with PTB [36]. Wang et al. found that pregnant women who slept for short periods at 8–16 weeks of gestation had infants with a significantly decreased birth length and birth weight [28]. Notably, this study found only maternal short sleep duration in early pregnancy was significantly associated with an increased risk of having an SGA infant (Table 2). Additionally, cross-over analysis (Table 4) indicated that sleep patterns that involved short duration during the early stage of pregnancy were associated with a higher risk of SGA. Therefore, we speculated that the first trimester or the period before the first trimester may represent a potential critical window during which maternal short sleep duration contributes to the risk of having an SGA infant. However, PSQI measured during early pregnancy may also reflect sleep duration before pregnancy; studies with a rigorous design are warranted to further determine the critical window for the contribution of short sleep duration to SGA risk.

### 4.2. Potential Mechanism for the Effect of Maternal Short Sleep Duration in Early Pregnancy on SGA

The mechanism by which short maternal sleep duration leads to SGA and why the early stage of pregnancy may represent a critical period for this association remains unclear but could be related to placenta insufficiency. We hypothesize that the placenta may play a role in this mechanism for the following reasons. It has been well documented that the placenta plays an essential role in delivering nutrients and oxygen from the mother to the fetus to maintain normal fetal growth. Further, dysfunctional placental development is responsible for many pregnancy complications, including fetal growth restriction. Additionally, the first trimester is a crucial period for the vasculature development of the placenta [37,38]. More importantly, several studies have found that sleep disorders can affect placental hypoxia, inflammation, and the secretion function of the placenta [39]. For example, it was found that disturbed sleep may interfere with normal placental bed vascular remodeling in early pregnancy, which could foster a chronic hypoxic environment and exert a negative impact on the placenta and fetal growth [40]. Okun et al. found that the concentration of IL-6 was significantly higher in the peripheral blood of pregnant women with short duration and poor efficiency of sleep [41]. Another case–control study reported lower levels of placenta associated plasma protein-A secreted by the placenta in pregnant women with obstructive sleep apnea [42]. Therefore, if maternal sleep only occurs in short periods during the first trimester of pregnancy, the vasculature development of the placenta may be seriously affected through the pathways mentioned above, resulting in the occurrence of SGA. Moreover, the process of pregnancy, including placental implantation and functional regulation and fetal growth, is largely regulated by the maternal endocrine system, which requires adequate sleep. Several other studies have reported that sleep deprivation could affect the regulation of the hypothalamic–pituitary–adrenal axis, resulting in the overexpression of cortisol, which is related to reduced fetal growth [43,44]. These might be the potential reasons for the first trimester of pregnancy being the critical period for maternal short sleep duration during pregnancy causing SGA.

### 4.3. Sex-Specific Effect of Maternal Sleep Quality and Duration in Early Pregnancy

Sex-specific effects of sleep disorder have been observed in previous studies. The results of the Shanghai Children Allergy Study showed that the significantly increased risk of childhood respiratory allergies associated with maternal short sleep duration, a lack of physical activity, and excessive screen exposure was only found in boys [45]. A prospective study including 3567 participants also found that the male babies of mothers who sleep less than 7 h had a greater reduction in birth length [28]. The subgroup analysis results in this study also suggested a sex-specific effect of sleep quality and duration in early pregnancy on SGA, with a significantly increased risk observed among boys but not among girls. This sex-specific effect could be explained by the different responses of the placenta and the yolk sac to maternal sleep disturbance. A study by Shah et al. showed that mothers carrying male babies who had poor sleep had placentas and yolk sacs with a significantly higher mean thickness and less uniformity. Vietheer and colleagues reported that maternal short sleep duration was related to larger yolk sacs for male embryos in early pregnancy to compensate for the adverse intrauterine environment [46]. Nevertheless, the exact pathway underlying the effects of sleep disorders on different sexes has not been elucidated, and the sex-specific association between sleep disorders and SGA needs to be considered in future investigations.

### 4.4. Strength and Limitations

To the best of our knowledge, this was the first prospective birth cohort study to exam the relationship between sleep quality and duration and the risk of having an SGA infant in three different periods of pregnancy. Within an ongoing birth cohort study, this study not only focused on sleep patterns during a specific stage of pregnancy but also took into account the quality and duration of sleep in early, middle, and late pregnancy to understand the impact of sleep on SGA comprehensively. Additionally, we adopted a more rigorous approach for participant selection and potential confounding variable adjustment to ensure the accuracy and reliability of the findings. Based on these advantages, this study provides new evidence and suggestions for pregnancy care, indicating that adequate sleep duration before or during the first trimester significantly reduces the risk of having an SGA infant. These findings are of great significance for prenatal management. However, the limitations should also be acknowledged. First, the sample size is relatively small, which limits the statistical power. For example, only mothers with both short sleep duration and poor sleep quality in early pregnancy are associated with higher risk of SGA in cross-over analysis, likely due to the small sample size in each group. Second, sleep quality and duration were assessed using the PSQI (a subjective measurement of sleep) rather than an objective measurement, which could have led to error reporting. Third, information on other specific sleep disorders, such as SDB, OSA, and insomnia, and psychiatric disorders, like stress and anxiety, as well as concomitant medications and nutrition status, were not collected in this study, though maternal BMI was adjusted in this analysis. It is possible that the association between sleep duration and the risk of having an SGA infant could be affected by SDB, OSA, or insomnia during pregnancy. Fourth, given the observational design, the possibility of potential residual confounding in analysis cannot be ruled out. Fifth, all the participants in this study were sampled from a single maternity and child healthcare hospital based in Shenzhen, China. Additionally, LGA was excluded in this study, which could limit the generalizability of our findings.

## 5. Conclusions

In summary, our findings suggest that maternal short sleep duration was associated with an increased risk of having an SGA infant and that the early stage of pregnancy might be the critical window for this association. Therefore, greater attention should be given to maternal sleep problems during pregnancy as part of antenatal care to help prevent SGA and improve birth outcomes.

## Figures and Tables

**Table 1:** Maternal characteristics and birth outcomes of 1677 participants in SZBCS.

Characteristic	Participants [n (%) or Mean (SD)]	AGA N = 1534	SGA N = 143	*p*
Demographics				
Maternal age (years)	30.54 (4.1)	30.63 (4.1)	29.52 (4.0)	0.002
Educational status				0.554
Senior high school or lower	198 (11.8)	189 (12.3)	13 (9.1)	
College or university	1252 (74.7)	1143 (74.5)	109 (76.2)	
Post-graduated or higher	227 (13.5)	206 (13.4)	21 (14.7)	
Income (CNY)				0.438
<CNY 5000	262 (15.6)	246 (16.0)	16 (11.2)	
CNY 5000–CNY 10,000	680 (40.5)	616 (40.2)	64 (44.8)	
CNY 10,000–CNY 15,000	409 (24.4)	373 (24.3)	36 (25.2)	
>CNY 15,000	326 (19.4)	299 (19.5)	27 (18.9)	
Marital status				0.953
Married	1617 (96.4)	1479 (96.4)	138 (96.5)	
Unmarried/widowed	60 (3.6)	55 (3.6)	5(3.5)	
Employment status				0.212
Unemployed	201 (12.0)	189 (12.3)	12 (8.4)	
Employed	1476 (88.0)	1345 (87.7)	131 (91.6)	
Health Status				
Pre-pregnancy BMI				0.005
Normal	1260 (75.1)	1168 (76.1)	92 (64.3)	
Underweight	290 (17.3)	252 (16.4)	38 (26.6)	
Overweight	127 (7.6)	114 (7.4)	143(9.1)	
Conception				0.577
Natural	1572 (93.7)	1440 (93.9)	132 (92.3)	
ART	105 (6.3)	94 (6.1)	11 (7.7)	
Parity				<0.001
Primiparous	951 (56.7)	840 (54.8)	111 (77.6)	
Multiparous	726 (43.3)	694 (45.2)	32 (22.4)	
Gestational hypertension				0.627
Yes	251 (14.4)	218 (14.2)	23 (16.1)	
No	1426 (85.6)	1316 (85.8)	120 (83.9)	
Gestational diabetes mellitus				0.624
Yes	16 (1.0)	14 (0.9)	2 (1.4)	
No	1661 (99.0)	1529 (99.1)	141 (98.6)	
Lifestyle				
Smoking before pregnancy				0.656
Yes	77 (4.6)	72 (4.7)	5 (3.5)	
No	1600 (95.4)	1462 (95.3)	138 (96.5)	
Alcohol intake before pregnancy				0.960
Yes	231 (13.8)	212 (13.8)	19 (13.3)	
No	1446(86.2)	1322 (86.2)	124 (86.7)	
Birth outcomes				
GA at delivery (weeks)	39.18 (1.0)	39.17 (1.0)	39.29 (1.1)	0.174
Birth weight (grams)	3227.64 (362.6)	3279.10 (284.7)	275.70 (220.48)	<0.001
Sex				0.807
Boys	890 (53.1)	816 (53.2)	74 (51.7)	
Girls	787 (46.9)	718 (46.8)	69 (48.3)	

CNY, Chinese yuan; BMI, body mass index; ART, assisted reproductive technology; GA, gestational age.

**Table 2:** Effect of maternal sleep quality and duration in different stages of pregnancy on SGA.

Sleep Type	Early Stage of Pregnancy				Mid-Stage of Pregnancy				Late Stage of Pregnancy		
	N	SGA	aOR (95%CI)		N	SGA	aOR (95%CI)		N	SGA	aOR (95%CI)
		[n (%)]				[n (%)]				[n (%)]	
Quality											
Good	908	72 (7.9)	1.00		999	84 (8.4)	1.00		918	75 (8.2)	1.00
Poor	769	71 (9.2)	1.29 (0.91~1.83)		678	59 (8.7)	1.06 (0.75~1.51)		759	68 (9.0)	1.14 (0.80~1.61)
Duration											
Normal	1258	94 (7.5)	1.00		1188	100 (8.4)	1.00		1087	92 (8.5)	1.00
Short	419	49 (11.7)	1.93 (1.32~2.79) ***		489	43 (8.8)	0.86 (0.59~1.28)		590	51 (8.6)	0.92 (0.64~1.33)

This model was adjusted for maternal age, pre-pregnancy body mass index (BMI), and parity. aOR: adjusted odds ratio. ***: *p* < 0.001.

**Table 3:** Combined effects of sleep quality and duration in different stages of pregnancy on risk of having SGA infant.

Sleep Type		Early Stage of Pregnancy				Mid-Stage of Pregnancy				Late Stage of Pregnancy		
Quality	Duration	N	SGA [n (%)]	aOR (95%CI)		N	SGA [n (%)]	aOR (95%CI)		N	SGA [n (%)]	aOR (95%CI)
Good	Normal	786	60 (7.6)	1.00		832	69 (8.3)	1.00		697	62 (8.9)	1.00
	Short	122	12 (10.7)	1.60 (0.79~3.02)		169	17 (10.1)	1.27 (0.69~2.22)		221	13 (5.9)	0.70 (0.36~1.26)
Poor	Normal	472	34 (7.2)	1.02 (0.65~1.57)		358	33 (9.2)	1.08 (0.69~1.68)		390	30 (7.7)	0.88 (0.55~1.38)
	Short	297	37 (12.5)	2.08 (1.32~3.23) ***		322	28 (8.7)	1.14 (0.70~1.81)		369	38 (10.3)	1.26 (0.81~1.93)

This model was adjusted for maternal age, pre-pregnancy body mass index (BMI), and parity. aOR: adjusted odds ratio. ***: *p* < 0.001.

**Table 4:** Effect of short sleep duration in stage-specific stages of pregnancy on SGA.

Short Sleep Duration in Pregnancy			N	SGA [n (%)]	aOR (95% CI)
Early Stage	Mid-Stage	Late Stage			
No	No	No	797	65 (8.2)	1.00
No	No	Yes	225	16 (7.1)	0.91 (0.49~1.57)
No	Yes	No	106	7 (6.6)	0.85 (0.35~1.81)
No	Yes	Yes	130	6 (4.6)	0.57 (0.22, 1.26)
Yes	No	No	99	9 (9.1)	1.38 (0.62~2.78)
Yes	No	Yes	67	10 (14.9)	2.08 (0.95~4.17)
Yes	Yes	No	85	11 (12.9)	1.92 (0.91~3.72)
Yes	Yes	Yes	168	19 (11.3)	1.82 (1.02~3.13) *

This model was adjusted for maternal age, pre-pregnancy body mass index (BMI), and parity. aOR: adjusted odds ratio. *: *p* < 0.05.

**Table 5:** Effect of maternal sleep quality and duration in different stages of pregnancy on SGA stratified by neonates’ sex.

	Sex	Early Stage of Pregnancy				Mid-Stage of Pregnancy				Late Stage of Pregnancy		
		N	SGA [n (%)]	aOR (95%CI)		N	SGA [n (%)]	aOR (95%CI)		N	SGA [n (%)]	aOR (95%CI)
Poor quality	Boys	429	44 (10.3)	1.79 (1.10~2.96) *		392	35 (8.9)	1.18 (0.72~1.91)		425	39 (9.2)	1.29 (0.80~2.10)
	Girls	340	27 (7.9)	0.94 (0.55~1.56)		286	24 (8.4)	0.96 (0.56~1.62)		335	29 (8.7)	1.01 (0.60~1.66)
Short duration	Boys	236	31 (13.1)	2.73 (1.63~4.56) ***		267	25 (9.4)	0.71 (0.43~1.21)		316	29 (9.2)	0.76 (0.47~1.27)
	Girls	183	18 (9.8)	1.33 (0.73~2.33)		223	18 (8.1)	1.06 (0.61~1.93)		374	22 (5.9)	1.09 (0.65~1.90)

This model was adjusted for maternal age, pre-pregnancy body mass index (BMI), and parity. *: *p* < 0.05; ***: *p* < 0.001.

## Data Availability

The data supporting the findings of this study are available on request from the corresponding author. The data are not publicly available due to privacy or ethical restrictions.

## Supplementary Materials

The following supporting information can be downloaded at: https://www.mdpi.com/article/10.3390/healthcare12232400/s1, Figure S1. Directed acyclic graph for the association between maternal sleep disturbance and SGA. Figure S2 Association of seven PSQI components in different stages of pregnancy with SGA. Table S1. Association of quartiles of maternal sleep quality and duration with SGA.
