# Supplementary material for: Association Between Sleep Quality and Duration During Pregnancy and Risk of Infant Being Small for Gestational Age: Prospective Birth Cohort Study
Source: Healthcare (Basel). 2024 Nov 29;12(23):2400. doi: 10.3390/healthcare12232400 (PMC11641115; doi:10.3390/healthcare12232400)
Supplement: Supplementary file 1 [file healthcare-12-02400-s001.zip › healthcare-3293278-supplementary.pdf]

Additional Material

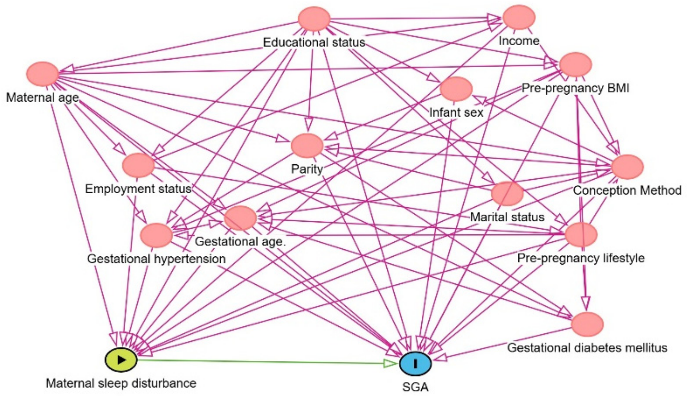

Figure S1 Directed acyclic graph for the association between maternal sleep disturbance and SGA.

BMI: pre-pregnancy body mass index, SGA: small for gestational age

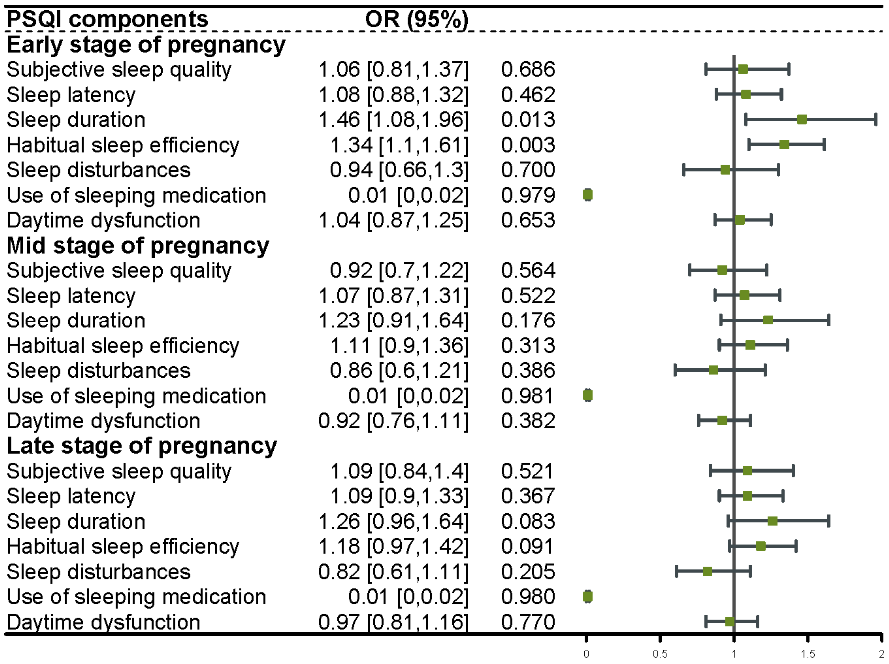

Figure S2 Association of seven PSQI components in different stage of pregnancy with SGA

PSQI: Pittsburgh Sleep Quality Index , SGA: small for gestational age.

Table S1 Association of quartiles of maternal sleep quality and duration with SGA

	Early pregnancy<br>aOR	Mid pregnancy<br>aOR	Late pregnancy<br>aOR
**Sleep quality**	*P for trend =0.084*	*P for trend =0.500*	*P for trend =0.101*
**Q1**	Ref	Ref	Ref
**Q2**	0.96 (0.56, 1.60)	1.20 (0.76, 1.90)	1.40 (0.85, 2.26)
**Q3**	1.11 (0.70, 1.74)	1.25 (0.77, 2.04)	1.05 (0.64, 1.69)

**Q4**	1.52 (0.96, 2.37)	1.16 (0.67, 2.00)	1.60 (1.01, 2.50) *
**Sleep duration**	*P for trend =0.009*	*P for trend =0.232*	*P for trend =0.090*
**Q1**	Ref	Ref	Ref
**Q2**	0.58 (0.39, 0.88) **	0.90 (0.61, 1.35)	0.70 (0.43, 1.17)
**Q3**	0.63 (0.34, 1.12)	0.62 (0.10, 2.19)	0.63 (0.34, 1.18)
**Q4**	0.48 (0.28, 0.79) **	0.75 (0.46, 1.21)	0.47 (0.17, 1.15)

This model was adjusted for maternal age, pre-pregnancy body mass index (BMI), parity. aOR: adjusted odd ratio. *: $p<0.05$; **: $p<0.01$; ***: $p<0.001$.
